# Genome-wide polygenic scoring for a 14-year long-term average depression phenotype

**DOI:** 10.1002/brb3.205

**Published:** 2014-02-12

**Authors:** Shun-Chiao Chang, M Maria Glymour, Stefan Walter, Liming Liang, Karestan C Koenen, Eric J Tchetgen, Marilyn C Cornelis, Ichiro Kawachi, Eric Rimm, Laura D Kubzansky

**Affiliations:** 1 Department of Social and Behavioral Sciences, Harvard School of Public HealthBoston, Massachusetts; 2 Channing Division of Network Medicine, Department of Medicine, Brigham and Women's Hospital, Harvard Medical SchoolBoston, Massachusetts; 3 Department of Epidemiology and Biostatistics, University of CaliforniaSan Francisco, California; 4 Department of Biostatistics, Harvard School of Public HealthBoston, Massachusetts; 5 Department of Epidemiology, Mailman School of Public Health, Columbia UniversityNew York, New York; 6 Department of Nutrition, Harvard School of Public HealthBoston, Massachusetts; 7 Department of Epidemiology, Harvard School of Public HealthBoston, Massachusetts

**Keywords:** Depression, GWAS, long-term cumulative phenotype, polygenic score, quantile regression

## Abstract

**Background:**

Despite moderate heritability estimates for depression-related phenotypes, few robust genetic predictors have been identified. Potential explanations for this discrepancy include the use of phenotypic measures taken from a single time point, rather than integrating information over longer time periods via multiple assessments, and the possibility that genetic risk is shaped by multiple loci with small effects.

**Methods:**

We developed a 14-year long-term average depression measure based on 14 years of follow-up in the Nurses' Health Study (NHS; *N* = 6989 women). We estimated polygenic scores (PS) with internal whole-genome scoring (NHS-GWAS-PS). We also constructed PS by applying two external PS weighting algorithms from independent samples, one previously shown to predict depression (GAIN-MDD-PS) and another from the largest genome-wide analysis currently available (PGC-MDD-PS). We assessed the association of all three PS with our long-term average depression phenotype using linear, logistic, and quantile regressions.

**Results:**

In this study, the three PS approaches explained at most 0.2% of variance in the long-term average phenotype. Quantile regressions indicated PS had larger impacts at higher quantiles of depressive symptoms. Quantile regression coefficients at the 75th percentile were at least 40% larger than at the 25th percentile in all three polygenic scoring algorithms. The interquartile range comparison suggested the effects of PS significantly differed at the 25th and 75th percentiles of the long-term depressive phenotype for the PGC-MDD-PS (*P *=* *0.03), and this difference also reached borderline statistical significance for the GAIN-MDD-PS (*P *=* *0.05).

**Conclusions:**

Integrating multiple phenotype assessments spanning 14 years and applying different polygenic scoring approaches did not substantially improve genetic prediction of depression. Quantile regressions suggested the effects of PS may be largest at high quantiles of depressive symptom scores, presumably among people with additional, unobserved sources of vulnerability to depression.

## Introduction

Depression is a complex disorder adversely affecting millions of individuals, with enormous social and economic costs (WHO 2001). The World Health Organization has predicted depression will be the second leading cause of disability worldwide by 2020 (Murray and Lopez 1996). Despite its public health importance, the biological mechanisms underlying the depression etiology remain uncertain.

Studies suggest that genetic factors play an important role in depression (Duffy et al. 2000), with a meta-analyzed heritability estimate from twin studies of 37% (95% confidence interval [CI], 31–43%) (Sullivan et al. 2000). However, it has been challenging to replicate previously identified candidate gene underlying susceptibility to depression (Lopez-Leon et al. 2008) or explain substantial variance in the phenotype. Genome-wide association studies (GWAS) have been unsuccessful in identifying significant individual genetic variants either (Sullivan et al. 2009; Lewis et al. 2010; Muglia et al. 2010; Shi et al. 2011; Shyn et al. 2011; Wray et al. 2012; Hek et al. 2013; Ripke et al. 2013). These negative findings have led to speculation that depression is particularly heterogeneous both clinically and etiologically, which could dramatically reduce statistical power to identify causal loci (Craddock et al. 2008). In addition, it is often hypothesized that for complex disorders including major depressive disorders, each individual risk allele only has low contribution, with odds ratio typically in the region of 1.05–1.2 (Mitchell 2012). Statistical power may be improved by increasing sample size, which is not always feasible; by improving precision of phenotype measurement; or by combining information from multiple loci such as creating polygenic scores (PS) or taking into account interactions between genes or loci, which may effectively increase the magnitude of the genetic effects.

Measurement has proven particularly challenging for depression-related phenotypes. Psychiatric research emphasizes distinctions between categorical diagnoses (binary phenotypes) and dimensional symptom measures (continuous phenotypes) (Maes et al. 1992; Prisciandaro and Roberts 2009). Because categorical diagnoses better distinguish individuals with true psychopathology, genetic determinants might be easier to identify when contrasting diagnosed cases to healthy controls. However, if the heritability reflects the independent influence of many genes with small effects, the phenotype is likely to be continuously distributed and more closely related to symptom-based measures. If so, using a binary outcome measure dichotomizing a continuous phenotype reduces statistical power compared to using a dimensional quantitative measure with the same sample size (Helzer et al. 2006; Kraemer 2007), In general, diagnostic and symptom-based measures are highly but not perfectly correlated (Radloff 1977).

A second challenge in measuring depression phenotypes is appropriately defining the time period of assessment. Genetic risks are carried throughout life, but the phenotype manifest at any given moment reflects both stable genetic contributions and fluctuating, transient contextual influences. These temporary variations reduce statistical power to detect genotype–phenotype associations (assuming transient contextual influences are independent of genetics). Depression phenotypes in genetic association studies are often assessed at a single time point when symptom measures are used. A registry-based twin study of depression suggested ongoing depression is more heritable than mild or nonrecurring depression using the diagnostic assessment Diagnostic and Statistical Manual of Mental Disorders, Third Edition (DSM-III) (Lyons et al. 1998). Integrating information across repeated assessments over time should reduce nongenetic variability in the phenotype and increase power to detect genetic determinants.

Combining information on multiple genetic determinants via polygenic scoring is another promising approach for explaining variance in complex phenotypes. PS combine information on many genetic variants, each presumed to have small effects, to predict phenotypes (Purcell et al. 2009). One application of PS combines information on candidate genes previously identified in the scientific literature. This pool is likely enriched with true causal loci, improving overall capacity to predict the phenotype. An alternative PS approach uses genome-wide data, adopting an agnostic prior regarding which alleles are causal and using more liberal *P*-value thresholds for selecting predictive polymorphisms compared to conventional criterion for genome-wide significance tests (Purcell et al. 2009). For some outcomes, it explained substantially more variance in the phenotype than scores limited to confirmed genotypes (Evans et al. 2009; Purcell et al. 2009). Demirkan et al. (2011) adopted this approach using the Genetic Association Information Network—Major Depressive Disorder (GAIN-MDD) sample to develop a genome-wide PS that explained up to 1% of the variance in depression.

We aimed to estimate the percentage of variance in a long-term average depression phenotype among participants in the Nurses' Health Study (NHS) that could be explained by PS using a genome-wide scan in NHS (NHS-GWAS-PS) or two external PS using weights derived by Demirkan et al. (GAIN-MDD-PS) or from the Psychiatric GWAS Consortium—Major Depressive Disorder (PGC-MDD-PS). We also briefly considered variance explained by a PS using candidate genes. On the basis of prior results from Demirkan's study, we anticipated that the PS could explain approximately 1% of the variance in the depression phenotype.

## Material and Methods

### Study participants

The NHS is a prospective cohort study of 121,700 U.S. female registered nurses aged 30–55 years at enrollment in 1976. Since then, self-administered questionnaires on medical history and lifestyle characteristics have been collected biennially. A subcohort of 32,826 women donated blood samples during 1989–1990. DNA was extracted from white blood cells using the QIAmpTM (Qiagen Inc., Chatsworth, CA) blood protocol and all samples were processed in the same laboratory. In the current analyses, we restricted to genetically defined unrelated white individuals with information on depression and genome-wide scan data available from four independent GWAS nested in NHS that passed quality control (QC) procedures (final analytic *N* = 6989). Details regarding study design and genotyping QC for each GWAS were reported elsewhere (Cornelis et al. 2011) and are summarized in Appendix 1 and Tables S2 and S3.

### Phenotype

Different assessments of depressive disorder or symptoms were collected in successive questionnaire cycles from 1992 to 2006 (Table S1), including standard symptom measures (e.g., CES-D [Center for Epidemiologic Studies Depression Scale]) and reports of antidepressant use or doctor-diagnosed depression. To combine information on depression across multiple sources of information over 14 years of follow-up, we derived a standardized composite depression score for each questionnaire cycle. We scaled depression measures at each wave to the Geriatric Depression Scale (GDS) administered in 2008, a depression symptom screening tool well-validated in the elderly (Sheikh and Yesavage 1986; Sharp and Lipsky 2002). We then used these scores to derive a 14-year long-term depression score representing average depression scores across all available questionnaire cycles through 2006 (up to seven waves). This phenotype captures more accurately both level and chronicity of depressive experience over time. More detailed description of the derivation of this measure is provided in Appendix 2^2010^.

To closely parallel previous study in GAIN-MDD by Demirkan et al. (2011), we also considered a dichotomized phenotype with the 14-year long-term depression score when applying GAIN-MDD-PS. To determine an appropriate cut-point, we dichotomized at the 89th percentile, which best corresponded to the cut-point of the CESD-10 symptom measure of depression (CESD-10 score ≥10) that is known to have optimal sensitivity and specificity for a major depressive disorder diagnosis (Andresen et al. 1994). A secondary analysis was also performed, comparing the long-term average depression score of individuals in the extremes: the lowest quartile versus the top 11th percentile.

In addition, we conducted another GWAS agnostic PS analysis using a second training set, a nine-GWAS-sample meta-analysis (which includes the GAIN sample) from the Psychiatric Genomics Consortium (PGC), which has been pruned to remove single-nucleotide polymorphisms (SNPs) in high linkage disequilibrium, and applied the weights and *P*-values in the PGC training set to the NHS samples. Similar to the procedure above, we first fit the continuous long-term composite depression score, then the dichotomous phenotype because the depression was originally analyzed as a dichotomous outcome in the PGC study.

### Genotyping and imputation

Exact QC protocols varied slightly by sample set (Tables S2 and S3). Individuals with genotyping completion or SNPs with call rates below 90% were excluded. Analyses based on principal components (Price et al. 2006) were conducted to assess race; any self-reported “white” samples that had substantial similarity to non-European reference samples were excluded. After QC, each study imputed to ∼2.5 million autosomal SNPs with NCBI build 36 of Phase II HapMap CEU data (release 22) as the reference panel using MaCH (Li et al. 2010) to account for the different genotyping platforms and the SNPs that failed to meet the QC criteria.

### Statistical analyses

#### Validation of the long-term average depression phenotype

We assessed construct validity by examining the association between the 14-year depression measure and established correlates of depression available in our sample: cigarette smoking (pack-years), physical activity (Mets per week), household characteristics, and phobic anxiety scale. We expected depression to be associated with greater likelihood of smoking, less physical activity, lower occupational and socioeconomic status, and higher degree of phobic anxiety. Details are described in Appendix 2^2010^.

#### Traditional GWAS

Genome-wide association analyses were first conducted separately for each NHS GWA substudies. A linear regression (using ProbABEL; Aulchenko et al. 2010) was performed on the long-term average depression score assuming additive genetic model, adjusting for age, disease status, and the top three or four principal components-derived eigenvectors to address residual population stratification (depending on the sample, as detailed in the Table S2). SNPs with minor allele frequency less than 2% or imputation quality of *R*^2^ less than 0.5 were excluded on a per-substudy basis. Meta-analysis using the METAL program was performed for each SNP across four NHS GWA substudies, combining allelic effects with inverse variance weighting (Willer et al. 2010). We used a genome-wide significance threshold *P* < 5 × 10^−8^. Our sample provides 80% power to detect a genetic effect size of 0.1 (corresponding to *R*^2^ of 0.006) with minor allele frequency of 0.15, under an additive genetic model.

#### Agnostic genome-wide polygenic scoring in NHS (NHS-GWAS-PS)

Genome-wide PS based on agnostic priors can provide a genetic risk score even when few of the causal genetic loci have been consistently identified in the literature. Following previously established methods, we first restricted to 1,584,339 SNPs with high imputation quality (*R*^2^ > 0.95) that were available across all four NHS GWA substudies. We next used the PLINK pruning procedure (200-SNP sliding window, pairwise *r*^2^ threshold of 0.25, and successive shift forward by five SNPs) to remove redundant SNPs, leaving a total of 97,883 independent SNPs.

Next, we performed a cross-validation procedure to obtain an unbiased estimate of the prediction performance. In the PS calculations, each time we used three of the four NHS GWA substudies as the “training” set to construct a polygenic risk score, which was then tested in the one remaining subsample (“testing” set). The procedure was conducted in three steps: (1) SNP-depression associations (beta weights) were first extracted from each of the three substudies in the training set. For each SNP, beta weights and *P*-values were meta-analyzed across the three substudies in the training set with GWAMA using inverse variance weights (Magi and Morris 2010); (2) the PS for each woman in the testing set was calculated as the sum of the number of risk alleles she carried at each locus meeting the selected *P*-value threshold, weighted by the meta-analyzed beta weight from the training set; and (3) we considered nine prespecified *P*-value thresholds in the training set for selecting SNPs to be included, ranging from 10^−5^ to 0.5 (*P*_training_). Alternatively, we also examined *P*_training_ thresholds with nonoverlapping ranges (10^−5^ < *P* < 10^−4^ to 0.4 < *P *<* *0.5) to assess whether any of these finer threshold groups explained more variance in depression. These PS were calculated using PLINK's SNP scoring routine. The cross-validation procedure was repeated four times, rotating the testing set each time (a “leave-one-out” procedure).

#### Genome-wide polygenic scoring from two external studies—GAIN-MDD (GAIN-MDD-PS) and PGC-MDD (PGC-MDD-PS)

In addition, we attempted to replicate the published finding by Demirkan et al. (2011). Through personal communication, Demirkan and colleagues provided the precise beta weights and *P*-values derived from their discovery set to facilitate replication in our cohorts. We also sought replication using data from another nine-study meta-analysis, which has been recently published (Ripke et al. 2013). We again considered the same nine *P*-value thresholds described above for selecting SNPs to be included in the PS calculation. The PS analysis was individually performed in each of the four NHS substudies and was meta-analyzed in the end.

#### Candidate gene polygenic scoring in NHS (candidate-PS)

Some investigators have suggested that the candidate gene approach is less likely to yield true causal loci, with most positive results arising by chance. In that case, a candidate gene approach to PS may exacerbate the difficulty of such efforts. However, given the significant literature on candidate genes, the ongoing controversies regarding which genes matter, and the substantial research attention they have received, the candidate gene polygenic scoring in NHS was also conducted and is described in detail in the Data S1. Briefly, to develop an informed candidate-PS, we selected 17 candidate genes with at least two positive prior reports of involvement in depression on the PubMed via the HuGE Navigator (Yu et al. 2008) as of May 2011. Ultimately 96 independent SNPs were reserved for analysis, and each candidate gene was represented by at least one SNP. We used the same cross-validation procedure to obtain an unbiased estimate of the prediction performance as described above.

#### Analyses of associations between PS and depression phenotypes

Assessments of the association between PS and depression phenotype were performed in *R* with linear (for continuous outcome) or logistic (for dichotomized outcome) regressions. Covariates included in all PS analyses were the same as in the SNP GWAS analysis: age, case–control status in each original GWAS, and the three or four eigenvectors. We further performed quantile regression models in the best prediction model of each approach defined by the significance level (NHS-GWAS-PS: *P *<* *0.2; GAIN-MDD-PS: *P *<* *0.001; and PGC-MDD-PS: *P *<* *0.2) to assess whether the effects of PS were larger at high levels of depression scores. Unlike linear regression models, which assess whether the mean value of the phenotype differs by PS level (the mean model), quantile regression models assess whether a specific percentile, for example, the median, differs by PS. Quantile regression was performed in the Statistical Analysis Systems software package, version 9.3 (SAS Institute, Inc., Cary, NC). Coefficients for each decile in each of the four GWA substudies were estimated and then meta-analyzed (with inverse variance weighting). We bootstrapped (5000 replications) to test the association between each of the three PS approaches and the interquartile range for the depression measure. A *P*-value <0.05 was considered a significant association with depression scores in quantile regressions.

## Results

### Initial analyses

The 14-year long-term average depression score of 6989 women in the study had a mean of 1.83 with standard deviation (SD) of 0.65, consistent with that in the full NHS cohort. The analytic sample did not appreciably differ from the larger cohort across a range of demographic and other sample attributes (Table 1).

**Table 1:** Characteristics of NHS full sample versus genetic study participants.

Characteristics	Full NHS sample	Genotyped sample
Sample size	121,701	6989
Age in 1992, mean (SD)	59.03 (7.28)	60.19 (6.72)
BMI in 1992, mean (SD)	26.22 (5.12)	26.70 (5.38)
Pack-year smoking in 1992, mean (SD)	13.54 (19.76)	13.26 (19.68)
Total activity in 1992 (Mets/week), mean (SD)	18.85 (23.18)	19.12 (21.74)
Age at blood draw, mean (SD)	—	57.79 (6.76)
Self-described Caucasian, *N* (%)	98,376 (94.5)	6797 (97.46)
Marital status in 1992, *N* (%)		
Married	72,454 (81.24)	5677 (82.78)
Widowed	9289 (10.42)	737 (10.75)
Divorced/separated	7416 (8.32)	439 (6.4)
Education, *N* (%)		
RN	62,673 (70.49)	4805 (70.21)
Bachelor degree	17,675 (19.88)	1369 (20.0)
Master degree	7726 (8.69)	609 (8.9)
Doctoral degree	834 (0.94)	61 (0.89)
Husband's highest education level, *N* (%)		
<High school graduate	4743 (6.34)	337 (5.66)
High school graduate	29,868 (39.92)	2408 (40.44)
College graduate	21,974 (29.37)	1686 (28.31)
Graduate school	18,242 (24.38)	1524 (25.59)
Father's occupation when participants were 16 years old, *N* (%)		
Blue collar	56,676 (51.66)	3071 (49.03)
White collar	42,788 (39.0)	2490 (39.76)
Farmer	10,248 (9.34)	702 (11.21)
14-year average depression score, mean (SD)	1.84 (0.62)	1.83 (0.65)
Missing 14-year average depression score, *N*	15,679	0
CESD-10 score, mean (SD)	5.72 (4.15)	5.76 (4.11)
Missing CESD-10 score, *N*	47,804	1093
CESD-10 ≥10, %	12.23	11.89

NHS, Nurses' Health Study; CESD, Center for Epidemiologic Studies Depression Scale—10 items.

**Figure 1:**
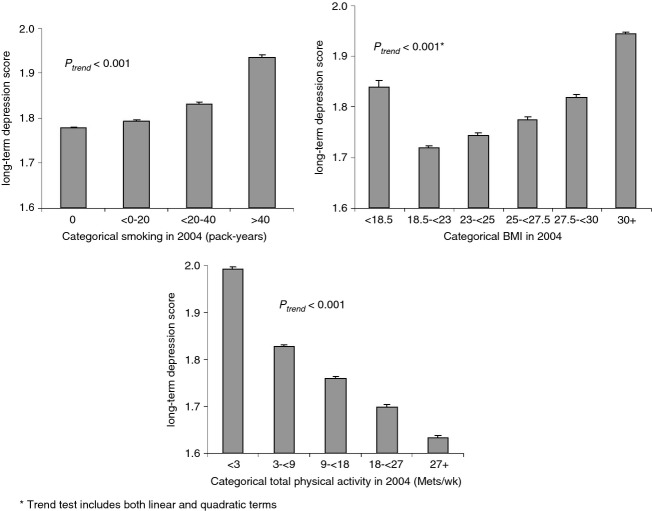
Distributions of behaviors and BMI in relation to the 14-year long-term average composite depression phenotype in the full NHS cohort (*N* = 106,020). BMI, body mass index; NHS, Nurses' Health Study.

The Cronbach's alpha for the seven-wave depression score was 0.83, suggesting these depression assessments measure a unified underlying attribute. In the full NHS cohort (*N* = 106,020), the long-term average depression score was significantly positively associated with cigarette smoking and negatively associated with physical activity (both *P*'s for trend <0.0001). The association between BMI and depression score was U-shaped (*P *<* *0.0001), such that both underweight and overweight women had higher depression scores than normal-weight women (Fig. 1).

### Meta-analyzed genome-wide SNP associations

**Figure 2:**
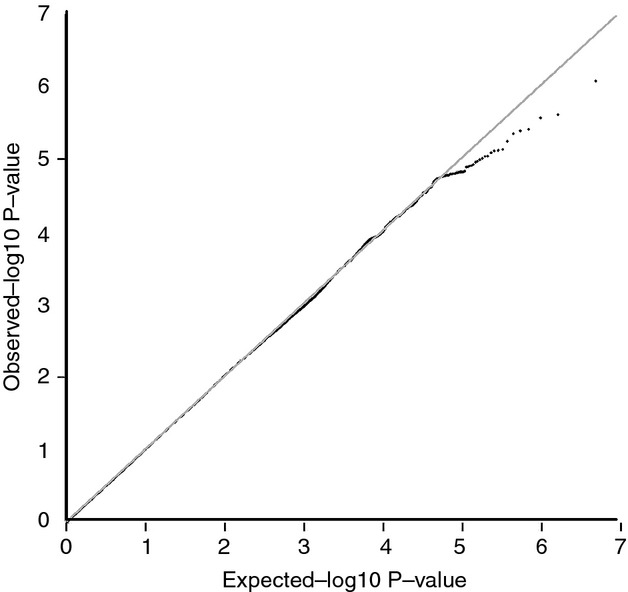
QQ plot of the GWAS meta-analysis in four NHS substudies (*N* = 6989). GWAS, genome-wide association studies; NHS, Nurses' Health Study.

**Figure 3:**
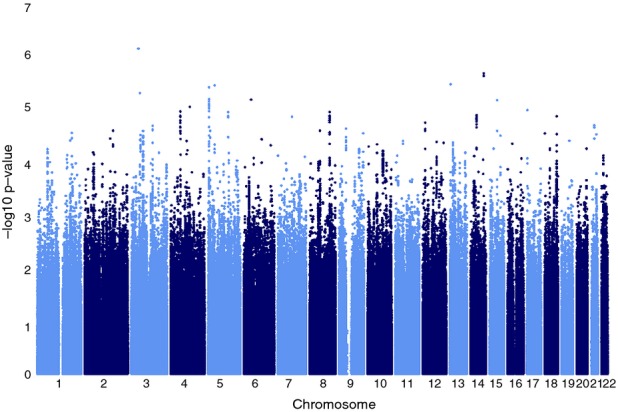
Manhattan plot of the GWAS meta-analysis in four NHS substudies (*N* = 6989). GWAS, genome-wide association studies; NHS, Nurses' Health Study.

The genomic inflation factor (lambda) for each substudy ranged between 1.00 and 1.01. The QQ-plot (Fig. 2) indicated good adherence of observed meta-analyzed *P*-values to the line of expectance, suggesting little evidence of systematic genotyping error. No individual SNPs reached the conventional genome-wide significance threshold of 5 × 10^−8^ for the association with long-term average depression score (Fig. 3). The SNP with the lowest *P*-value was rs6763048 (*P *=* *8.42 × 10^−7^), mapping to an intron of SCN5A on chromosome 3. A total of 14 SNPs had *P*-values <1 × 10^−5^, corresponding to eight independent SNPs (*r*^2^ < 0.05 in 500 kb) (Table 2).

**Table 2:** Meta-analysis GWAS results of 14-year long-term average composite depression measure of top findings (*P* < 10^−5^) in four NHS substudies (*N* = 6989).

SNP	Chr	Position	Allele 1/Allele 2	Allele 1 frequency	Closest gene	Approx. distance (kb)	Effect	Standard error	*P*-value
rs6763048	3	38656398	A/G	0.858	*SCN5A*	Intron	−0.076	0.016	8.42E-07
rs9323902^1^	14	93641863	T/G	0.257	*IFI27L1*	3	−0.063	0.013	2.43E-06
rs10873447^1^	14	93642502	A/T	0.748	*IFI27L1*	3.6	0.063	0.014	2.71E-06
rs4366580	13	22333062	T/C	0.200	*IPMKP1*	23	−0.062	0.013	3.90E-06
rs10512653	5	36474011	T/C	0.150	*RANBP3L*	136	0.069	0.015	4.08E-06
rs252928^2^	5	5564990	A/G	0.345	*KIAA0947*	21	0.052	0.011	4.43E-06
rs17287770	3	46629429	C/G	0.928	*LOC100132146*		−0.096	0.021	5.69E-06
rs2619855^2^	5	5557842	T/C	0.653	*KIAA0947*	14	−0.051	0.011	7.40E-06
rs4266492	6	40803132	A/C	0.950	*LRFN2*	139	0.137	0.031	7.56E-06
rs7182961	15	60707884	C/G	0.749	*TLN2*	19	0.057	0.013	7.70E-06
rs252930^2^	5	5562703	A/C	0.339	*KIAA0947*	19	0.051	0.011	8.15E-06
rs252929^2^	5	5563450	T/C	0.339	*KIAA0947*	20	0.051	0.011	9.13E-06
rs2652715^2^	5	5567517	A/G	0.659	*KIAA0947*	24	−0.051	0.011	9.16E-06
rs2578514^2^	5	5574830	A/G	0.651	*KIAA0947*	31	−0.050	0.011	9.81E-06

GWAS, genome-wide association studies; NHS, Nurses' Health Study; SNP, single-nucleotide polymorphism.

1 SNPs in linkage disequilibrium with each other (*r*^2^ > 0.8) on chromosome 14.

2 SNPs in linkage disequilibrium with each other (*r*2 > 0.8) on chromosome 5.

### NHS-GWAS-PS analyses

The genome-wide PS similarly explained a small fraction of variance in the long-term average depression score (Table 3). Using the most liberal threshold of *P *<* *0.5 to select SNPs in the training set, the genome-wide PS was associated with the depression score in the testing set (*P *=* *0.004), but explained only 0.1% of the variance. The maximum percentage of variance explained was achieved with slightly more conservative *P*-value thresholds for SNP selection (at *P *<* *0.3), in which the genome-wide PS explained 0.2% of the variance (*P *=* *0.003). When restricted to nonoverlapping *P*_training_ threshold ranges, the SNPs with the most significant association were those with *P*_training_ between 0.1 and 0.2; this group alone comprised nearly 9900 SNPs, but explained 0.1% of phenotype variation (Table 3).

**Table 3:** Meta-analysis of percentage of variance explained in depression phenotype in NHS by the genome-wide agnostic polygenic scores in the leave-one-substudy-out analysis (*N* = 6989).

Cumulative *P*-value thresholds for selecting SNPs			Nonoverlapping *P*-value thresholds for selecting SNPs		
*P*_training_^1^ threshold	Percentage of variance explained	*P*-value	*P*_training_^1^ threshold	Percentage of variance explained	*P-*value
p<0.00001	0.1	0.355	0–0.00001	0.1	0.355
p<0.0001	0	0.528	0.00001–0.0001	0	0.944
p<0.001	0.2	0.159	0.0001–0.001	0.2	0.118
p<0.01	0.1	0.524	0.001–0.01	0.1	0.977
p<0.1	0.1	0.043	0.01–0.1	0.1	0.013
p<0.2	0.1	0.002	0.1–0.2	0.1	0.003
p<0.3	0.2	0.003	0.2–0.3	0.1	0.832
p<0.4	0.1	0.002	0.3–0.4	0.1	0.269
p<0.5	0.1	0.004	0.4–0.5	0	0.996

NHS, Nurses' Health Study; SNP, single-nucleotide polymorphism.

1 Training set: remaining three NHS substudies except the testing sample.

### GAIN-MDD-PS and PGC-MDD-PS analyses

Regardless of the *P*-value threshold chosen, the GAIN-MDD-PS was not significantly associated with either the continuous or dichotomized depression phenotype in the NHS sample (Table 4). The maximal proportion explained by genome-wide PS comparing women at the extremes of the phenotype was higher than that in the full sample (0.4% vs. 0.1%); however, it was not statistically significant, likely due to the reduction in sample size when using only individuals with extreme values of the phenotype (*n* = 2920) (data not shown).

**Table 4:** Meta-analysis of percentage of variance explained in depression phenotype in NHS by the genetic risk scores using external GAIN-MDD sample as the training set (*N* = 6989).

Outcome in NHS testing set				
	Continuous depression score		Dichotomous depression status	
*P*_training_ threshold	*R*^2^%^1^	*P*-value^1^	*R*^2^%^1^, ^2^	*P*-value^1^
p<0.00001	—	—	—	—
p<0.0001	0.1	0.807	0.1	0.697
p<0.001	0.1	0.203	0.2	0.450
p<0.01	0	0.866	0	0.775
p<0.1	0	0.922	0.1	0.520
p<0.2	0	0.581	0	0.863
p<0.3	0	0.394	0.1	0.903
p<0.4	0	0.300	0.1	0.651
p<0.5	0.1	0.344	0.1	0.653

GWAS, genome-wide association studies; GAIN-MDD, Genetic Association Information Network—Major Depressive Disorder; NHS, Nurses' Health Study.

1 Use the GWAS result from GAIN-MDD as the training set, and use each of the four NHS substudies as the testing set on recurrent composite depression score. The final weighted *R*^2^ and *P*-value calculated meta-analytically across four NHS substudies.

2 Denotes Nagelkerke's *R*^2^%.

When applying the agnostic PS from a nine-study meta-analysis of PGC-MDD, the genome-wide risk scores derived from SNPs with less stringent *P*_training_ threshold were significantly associated with the continuous long-term depressive score, but they only explained at most 0.1% of variance in phenotype. The Nagelkerke's *R*^2^ was also at most 0.1% when the depression phenotype was modeled dichotomously without the statistical significance (Table 5).

**Table 5:** Meta-analysis of percentage of variance explained in depression symptoms in NHS by the genetic risk scores using external PGC-MDD sample as the training set (*N* = 6989).

Outcome in NHS testing set				
	Continuous depression score		Dichotomous depression status	
*P*_training_ threshold	*R*^2^%^1^	*P*-value^1^	*R*^2^%^1^, ^2^	*P*-value^1^
p<0.00001	0	0.445	0.1	0.951
p<0.0001	0.1	0.192	0.1	0.309
p<0.001	0	0.644	0.1	0.291
p<0.01	0	0.240	0	0.639
p<0.1	0.1	0.078	0.1	0.309
p<0.2	0.1	0.030	0.1	0.263
p<0.3	0.1	0.049	0.1	0.46
p<0.4	0.1	0.041	0.1	0.415
p<0.5	0.1	0.031	0.1	0.313

GWAS, genome-wide association studies; PGC-MDD, Psychiatric GWAS Consortium—Major Depressive Disorder; NHS, Nurses' Health Study.

1 Use the nine-study meta-analyzed GWAS result from PGC-MDD as the training set, and use each of the four NHS substudies as the testing set on long-term composite depression score. The final weighted *R*^2^ and *P*-value calculated meta-analytically across four NHS substudies.

2 Denotes Nagelkerke's *R*^2^%.

### Candidate-PS analyses

Three individual SNPs (rs36011, rs1417584, and rs6917735) showed nominally significant associations at α threshold of 0.05, but none remained significant after Bonferroni correction. Overall, the candidate-PS explained a small fraction of the variance in the long-term average depression scores in the NHS leave-one-out meta-analysis (see Data S1).

### Quantile regression analyses

**Figure 4:**
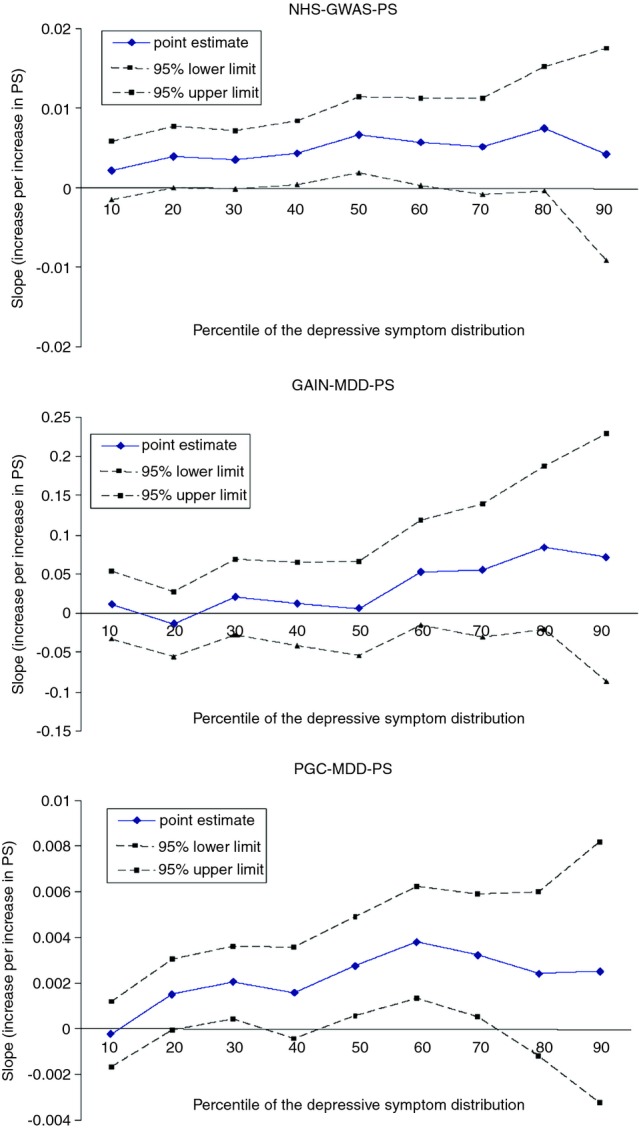
Quantile plot of polygenic scores (PS) on 14-year long-term average composite depression phenotype.

The quantile regression suggested a modest increase in PS effect on depression score in higher quantiles than in lower quantiles (Fig. 4). The pseudo-*R*^2^ increased more than 40% in the 75th percentile quantile regression model compared to that in the 25th percentile model in all three PS approaches. The interquartile range comparison suggested the effects of PS significantly differed at the 25th and 75th percentiles of the long-term depressive phenotype for the PGC-MDD-PS (*P *=* *0.03) (pseudo *R*^2^ changed from 0.1% at the 25th percentile to 0.3% at the 75th percentile), and this difference was at borderline statistical significance for the GAIN-MDD-PS (*P *=* *0.05). The result of candidate gene polygenic scoring could be found in the Table S5.

## Discussion

In this sample of 6989 women, we did not identify any SNPs significantly associated with a 14-year average composite depression phenotype using either candidate gene-based or conventional GWAS analyses. With the two approaches that developed PS (NHS-GWAS-PS and PGC-MDD-PS), we achieved nominal statistical significance, but never explained more than 0.2% of the phenotypic variance. While the PS analyses indicated that SNPs with *P*-values above conventional significance thresholds may contribute to the association, the proportion of variance explained was much smaller than that reported in a prior study (0.2% vs. 1%) (Demirkan et al. 2011). Furthermore, the GAIN-MDD-PS did not predict depression in our mean model analyses. The quantile regression results suggested modestly larger effects of PS on high- versus low- depression quantiles, but even at high depression quantiles (e.g., 75% percentile), the PS explained at most 0.3% of phenotype variance.

Our findings are in line with the literature in which no locus surpassed genome-wide significance in relation to depression (Sullivan et al. 2009; Lewis et al. 2010; Muglia et al. 2010; Shi et al. 2011; Shyn et al. 2011; Wray et al. 2012; Hek et al. 2013; Ripke et al. 2013). Of note is that in a largest GWAS of psychiatric illness to date (with *N* over 60,000), the PGC Cross-Disorder Group identified SNPs at four loci that were significantly associated with a cross-disorder phenotype as identified by meta-analyzing across five childhood-onset and adult-onset psychiatric disorders including major depressive disorder, bipolar disorder, schizophrenia, autism spectrum disorders, and ADHD, and using a goodness-of-fit model-selection procedure (Cross-Disorder Group of the Psychiatric Genomics Consortium 2013). Findings suggest the potential for shared genetics between these psychiatric disorders. However, because the heritability estimate of depression alone is modest, attempts to identify disease-specific susceptibility loci are expected to be challenging. Moreover, associations derived from twin studies include heritability directly attributable to genes and to gene by shared environment interactions. As a result, inability to identify relevant environments and gene–environment interactions is likely to reduce success when searching for depression susceptible genes. It is further possible that relevant genetic factors are due to private or rare mutations not captured by GWAS chips or expression variations such as epigenetics; this could also explain why our PS explained little variation in the depression phenotype.

Consistent with previous research, our findings suggest each common genetic variant of depression has a very small effect and therefore is difficult to detect. We anticipated that the aggregate risk combining information on multiple loci would strengthen our explanatory capacity. This was supported in that the PS significantly predicted long-term average depression score, but the improvement was an order of magnitude smaller than necessary to explain the missing heritability. The limited explanatory power of the genome-wide PS should be interpreted cautiously because such agnostic PS are likely composed primarily of false positives. Thus, the genome-wide PS may include a few true causal loci plus thousands of unrelated loci; adding substantial noise to any causal variable will inevitably reduce its correlation with the outcome. The explanatory power of the genome-wide PS is likely to increase with larger sample sizes, as the ratio of true to false positives improves.

We also improved on prior GWA studies by using a dimensional phenotype summarizing depressive symptoms over 14 years. The literature suggests the etiology of depression involves multiple genes each with small effect, thus the relevant phenotype is likely to be normally distributed. In addition, the long-term average score is enhanced by virtue of having both valid symptom measures (Radloff 1977; Silveira et al. 2005) and direct information about depression diagnoses. This phenotype should be less influenced by transient environmental factors and therefore more strongly related to stable genetic predispositions. The enhanced phenotype was not strongly predicted by the PS, however, suggesting the use of cross-sectional depression phenotypes is not the critical barrier to identifying genetic determinants. On the other hand, as depression is suspected to be a heterogeneous phenotype, in which individual patients may have a wide range of clinical manifestations and simultaneously develop comorbid disorders, identifying a depression-related phenotype which captures more homogeneous clinical features may be critical for identifying the underlying genetic architecture. Prior research has attempted to index plausible sources of phenotypic heterogeneity in the depression cases by stratifying analyses by gender, recurrence, age of onset, or typicality, but such efforts have not yielded statistically significant findings. This study only included female subjects, and incorporated depression-related phenotype information collected multiple times across 14 years; as a result our phenotype may capture a more chronic attribute or experience. However, age of onset was not available in this study and the cases had a mixture of symptom severity. The possible phenotypic heterogeneity, if likely linked to genetic heterogeneity, could reduce statistical power to detect association signals.

We found heterogeneous PS effects across quantiles of depression, consistent with the hypothesis that some loci have worse effects on individuals with other types of environmental or genetic vulnerability (Williams 2012). Because we use a genome-wide PS, environmental factors such as adverse life events or lack of social support seem most likely. The larger effect of PS on high- versus low- depression quantiles may support the hypothesis that the “missing heritability” is attributable to epistatic or environmental interactions, such that some genotypes are relevant only in the context of other risk factors. Nearly all twin studies rely on twins raised together; in such studies, the variance attributable to shared environmental factors modifying genetic effects is implicitly included in heritability estimates (Kamin and Goldberger 2002). Gatz et al. (1992) found little additive genetic variance among twin pairs reared apart, suggesting the likely importance of environment and gene–environment interactions. Alternatively, heterogeneous PS effects across quantiles of the phenotype might represent noninterval scaling of the phenotype or modeling error. Regardless of whether the result is interpreted as evidence for gene–environment interactions, the finding of heterogeneous effect sizes indicates that mean effects estimated in linear regression model may understate the overall impact of genetic risk.

Potential limitations of our study include generalizability of the NHS blood sample, imprecision in depression assessment, and different GWA platforms available in each subcohort. Combing multiple GWAS results across cohorts with different genotyping platforms and QC filters is now common when studying the genetics of complex diseases such as depression and schizophrenia, because large sample sizes are necessary (Schizophrenia Psychiatric Genome-Wide Association Study Consortium 2011; Hek et al. 2013). The QC has been carefully and extensively examined internally, and the allele frequencies are similar across NHS subcohorts.

In summary, combining longitudinal phenotype assessments from multiple measurements and different polygenic scoring approaches did not substantially improve genetic prediction of depression. Common SNPs explained 0.2% or less of depression variance via polygenic scoring analysis. Many studies now suggest depression does not result from either purely genetic or environmental influences, but rather from the intersection of the two (Dunn et al. 2011). Because both components are intertwined, the underlying association could be obscured if either factor is neglected, especially when the environments differ widely between samples. Identifying social or environmental modifiers of genetic risks is a critical next step to understand depression etiology.

## Appendix 1

### Nested Genetic Case–Control Studies in NHS

Participants for the current analyses were from four independent GWAS nested in the NHS initially designed to study outcomes of breast cancer (BrCa, *N* = 2280), coronary heart disease (CHD, *N* = 1135), type 2 diabetes (T2D, *N* = 3084), and kidney stone (KS, *N* = 490) disease. Both cases and controls in each original GWAS were included for analysis.

Briefly, for the NHS BrCa study, cases and controls were limited to postmenopausal women. Controls were matched with cases by age and postmenopausal hormone use at blood draw. For the NHS CHD study, controls were randomly selected from the subcohort who provided blood samples and did not experience CHD with two controls for each case, matched with cases by age, smoking, and month of blood draw. For the NHS T2D study, controls who were defined to be those free of diabetes at the time the case was reported were matched on year of birth, month of blood collection, and fasting status. For the NHS KS study, participants with a history of kidney stones were matched to randomly selected controls identified in two cycles from those with no history of cancer (cycles 1 and 2) or cardiovascular disease (cycle 1) who met age eligibility requirements (cycle 1: <66; cycle 2: <76). A total of 2280 women from the NHS BrCa substudy, 1135 women from the CHD substudy, 3084 women from the T2D, and 490 women in the KS substudy, all unrelated and genetically defined whites who had nonmissing phenotype and covariate data, were included in this study (total *N* = 6989).

## Appendix 2

### Construction of a 14-Year Long-Term Average Depression Measure

The Nurses' Health Study (NHS) began collecting depression-related measures starting in 1992. Information on clinical and subclinical levels of depression has been assessed with a variety of measures across seven subsequent interview waves. For example, information on clinician-diagnosed depression has been assessed every 2 years since 2000, and information on antidepressant use has been collected every 2 years since 1996. Questionnaire-based measures with relevant symptom items were also administered, including the SF-36, Center for Epidemiologic Studies Depression Scale—10 items (CESD-10), life orientation test, and geriatric depression scale—15 items (GDS-15). These have been assessed either once or at multiple points (Table S1). For the construction of a 14-year long-term average phenotype, all assessments were scaled against a common anchor score to make it possible to combine measures across waves. The GDS, assessed in 2008, has excellent psychometric characteristics within the age span of our population, has good validity as a continuous dimensional measure of depressive symptomology, and good sensitivity and specificity for clinical depression when dichotomized (Sheikh and Yesavage 1986). We therefore chose the GDS as our “anchor” assessment and scaled all other assessments against the GDS. We chose GDS-15 over CESD-10 because it was specifically developed for use in geriatric population (the mean age of NHS participants were both over 70 years old when either instrument was examined), it contained fewer somatic items. In cognitively intact patients older than 65 years, the GDS screen is the preferred instrument because the psychometric data on the CES-D are mixed in this population (Sharp and Lipsky 2002). Although the quality of the available measures used across waves differs, our approach down-weights those instruments that do not correspond well with the GDS. Our protocol was as follows: using all NHS women with GDS scores (regardless of the availability of genetic data), we regressed the GDS score on all depression-related measures available in that wave, using a linear regression model. For example, using all measures of the depression phenotype available in 2004, we estimated the following linear regression:

(1)

On the basis of this linear regression, we predicted the value that the GDS score would have taken if it has been assessed in 2004. We estimated similar models for each interview wave, 1992–2006. For instruments with missing data on a few items, we used the average of nonmissing items if at least half of the items were reported and a missing indicator method for observations missing more than half of the items.

In a second step, we used the regression coefficients from the initial models to predict the value of GDS for each participant at each wave, had he or she been given the GDS. In this way, all individual depression measures collected at one wave were rescaled and translated to a single common scale (GDS-standardized score) for each participant, and these estimates could be obtained even for individuals who did not complete the GDS in 2008. The final phenotype was the average of the rescaled depression scores from all available questionnaire cycles (up to seven waves):

(2)

This approach maximized the available sample size and optimized the information available on lifetime experiences of depression, because anyone with at least one wave of information with depression assessment was included. We also believe it decreased the transient component of the measure compared to using a single-wave assessment, which would strengthen our ability to detect genetic predictors. In fact, in our analytic sample 132 (1.9%) women had only one measure of depression, 136 (1.9%) had two measures, and 6721 (96.2%) had three or more measures, in which 5256 (78%) had complete data collection of depression measures in all waves.

We assessed reliability and validity of the 14-year long-term average composite depression phenotype in the full NHS sample. First, we examined the correlation of the standardized measures across waves with a commonly used measure of depressive symptoms, the CESD-10 assessed at a single-wave in 2004. For the 73,897 women with both CESD-10 and the 14-year average depression measure, the Pearson correlation coefficient was 0.74. This high but not perfect correlation suggests that the 14-year average phenotype may have more information in it. We assessed construct validity by examining the association between the 14-year composite measure and established correlates of depression: cigarette smoking (pack-years) and physical activity (Mets per week), assessed by self-report in 2004; paternal occupation when participants were 16 years old in 1976, husband's education as a measure of socioeconomic status assessed in 1992, and the average of phobic anxiety scale of the Crown Crisp Experimental Index (CCI) between 1988 and 2004. We expected the new 14-year average depression score to be associated with greater likelihood of smoking, less physical activity, and lower occupational and socioeconomic status (Table 1). We also anticipated that 14-year average depression would be more correlated with these factors than a depressive symptom measure at any single time point.

The mean (SD) of the new 14-year average composite depression score in women with depression defined by CESD-10 was 2.68 (0.68), significantly higher than those without elevated CESD-10 scores (mean = 1.66, SD = 0.47, *t*-test *P*-value <0.0001). As expected, cigarette smoking, physical activity, and household characteristics including husband's highest education and paternal occupation when participants were 16 years old, and phobic anxiety scale in CCI all explained slightly more variance in our 14-year average depression score than they explained for the 2004 CESD-10 score (Table S4). This result again suggests that the 14-year average depression measure captures more information about a stable phenotype than the single-wave measure alone.

## Supporting Information

**Data S1.** Candidate Gene Polygenic Scoring in NHS (NHS-Candidate-PS) **Figure S1.** Quantile plot of polygenic scores (PS) on 14-year long-term average composite depression phenotype. **Table S1.** Depression-related measures collected in the Nurses' Health Study. **Table S2.** Study-specific genotyping, imputation, and statistical analysis. **Table S3.** Sample quality control. **Table S4.** Percentage of variance* in depression phenotypes explained by variables associated with depression in the NHS full cohort, restricting to women with nonmissing 2004 CESD-10 and 14-year long-term average depression scores (N = 73,897). **Table S5.** Meta-analysis of percentage of variance explained in depression phenotype in NHS by the candidate gene polygenic scores in the leave-one-substudy-out analysis (N = 6989).
