# Supplementary material for: Genome-wide polygenic scoring for a 14-year long-term average depression phenotype
Source: Brain Behav. 2014 Feb 12;4(2):298–311. doi: 10.1002/brb3.205 (PMC3967544; doi:10.1002/brb3.205)
Supplement: Supplementary file 1 — Data S1. Candidate Gene Polygenic Scoring in NHS (NHS-Candidate-PS) Figure S1. Quantile plot of polygenic scores (PS) on 14-year long-term average composite depression phenotype. Table S1. Depression-related measures collected in the Nurses' Health Study. Table S2. Study-specific genotyping, imputation, and statistical analysis. Table S3. Sample quality control. Table S4. Percentage of variance* in depression phenotypes explained by variables associated with depression in the NHS full cohort, restricting to women with nonmissing 2004 CESD-10 and 14-year long-term average depression scores (N = 73,897). Table S5. Meta-analysis of percentage of variance explained in depression phenotype in NHS by the candidate gene polygenic scores in the leave-one-substudy-out analysis (N = 6989). [file brb30004-0298-sd1.docx]

*Candidate Gene Polygenic Scoring in NHS (NHS-Candidate-PS)*

To develop an informed candidate-PS, we selected 17 candidate genes with at least two positive prior reports of involvement in depression on the PubMed via the HuGE Navigator [[33](#_ENREF_33)] as of May 2011: *APOE*, *MTHFR*, *AVPR1B*, *DISC1*, *HTR1A*, *NR3C1*, *TNF*, *FKBP5*, *TPH1*, *BDNF*, *GNB3*, *TPH2*, *P2RX7*, *HTR2A*, *SLC6A2*, *SLC6A4*, and *MAOA*. These were matched to the imputed SNPs by gene transcript position +/- 20kb using the UCSC genome browser [[34](#_ENREF_34)] with the NCBI36/hg18 assembly. From 1,710 SNPs in candidate gene regions, we then restricted to 1,007 SNPs with high imputation quality of r^2^ > 0.95 in all four NHS substudies. *AVPR1B* and *APOE* were then dropped due to no SNPs in the region or failure to meet imputation quality threshold. We next used the PLINK pruning procedure (200-SNP sliding window, pairwise r^2^ threshold of 0.25, and successive shift forward by 5 SNPs) to remove redundant SNPs. Ultimately 96 independent SNPs were reserved for analysis, and each candidate gene was represented by at least 1 SNP.

This SNP panel of 96 independent SNPs was used to derive a NHS-candidate-PS using the same cross-validation procedure described in the main text. In the PS calculations, each time 3 of the 4 NHS GWA substudies were designated as the “training” set to construct a polygenic risk score which was then tested in the one remaining subsample (“testing” set). The procedure was conducted in 3 steps: (1) SNP-depression associations (beta-weights) were first extracted from each of the 3 substudies in the training set. For each SNP, beta-weights and p-values were meta-analyzed across the 3 substudies in the training set with GWAMA using inverse variance weights [[33](#_ENREF_33)]; (2) The PS for each woman in the testing set was calculated as the sum of the number of risk alleles she carried at each locus meeting the selected p-value threshold, weighted by the beta weight from the training set; (3) We considered 9 pre-specified p-value thresholds in the training set for selecting SNPs to be included, ranging from 10^-5^ to 0.5 (P_training_). We also examined P_training_ thresholds with non-overlapping ranges (10^-5^<*p*<10^-4^ to 0.4<*p*<0.5) to assess whether any of these finer threshold groups explained more variance in depression. These PS were calculated using PLINK's SNP scoring routine. The cross-validation procedure was repeated four times, rotating the testing set each time.

The candidate-PS explained a small fraction of the variance in the long-term average depression scores in the NHS leave-one-substudy-out meta-analysis (Supplementary Table S5). For example, the candidate-PS (with p-value threshold <0.5 in the training set) predicted the depression at *p*=0.001, with an R^2^ of 0.1% (Supplementary Table S5, bottom row). Using slightly more conservative *p*-value thresholds for PS SNP selection improved the score only slightly; the maximum variance explained was with a *p*-value<0.3 threshold, at which 0.2% of the variance in the depression score was explained. Using P_training_ thresholds with non-overlapping ranges suggested that SNPs with P_training_ ranging between 0.1 and 0.2 were the strongest contributors to the candidate gene PS.

For consistency with the primary analyses, we further performed quantile regression models in the best prediction model defined by the significance level (NHS-candidate-PS: *p*<0.3) to assess whether the effects of candidate gene PS were larger at high levels of depression scores. The interquartile range comparison suggested the effects of polygenic scores differed (with borderline statistical significance) between the 25^th^ and 75^th^ percentiles for the NHS-candidate-PS (*p*=0.07).

**Supplementary Table S1. Depression-related measures collected in the Nurses’ Health Study**

| Year | 1992 | 1994 | 1996 | 1998 | 2000 | 2002 | 2004 | 2006 | 2008 |
| --- | --- | --- | --- | --- | --- | --- | --- | --- | --- |
| Clinician-diagnosed depression |  |  |  |  | X | X | X | X | X |
| Depression medication use |  |  | X | X | X | X | X | X | X |
| SF-36 mental health |  |  |  |  |  |  |  |  |  |
| Role emotional scale | X |  | X |  | X |  |  |  |  |
| Social function scale | X |  | X |  | X |  |  |  |  |
| MHI-5 | X |  | X |  | X |  |  |  |  |
| Vitality scale | X |  | X |  | X |  |  |  |  |
| CESD-10 |  |  |  |  |  |  | X |  |  |
| Life orientation test |  |  |  |  |  |  | X |  | X |
| Geriatric Depression Scale – 15 items (GDS-15) |  |  |  |  |  |  |  |  | X |
| Additional items |  |  |  |  |  |  |  |  |  |
| Felt hopeless about the future |  |  |  |  | X |  |  |  |  |
| Thought about or wanted to commit suicide |  |  |  |  | X |  |  |  |  |
| Felt no interest in things |  |  |  |  | X |  |  |  |  |
| Difficulty falling asleep or staying asleep |  |  |  |  | X |  |  |  |  |
| Felt sad, blue, or depressed for most of the day  nearly every day in two weeks or longer in life time |  |  |  |  |  |  | X |  |  |

**Supplementary Table S2. Study-specific genotyping, imputation and statistical analysis**

| **Study** | **Genotyping** | | | | | **Imputation** | | | **Association Analysis** | | | |
| --- | --- | --- | --- | --- | --- | --- | --- | --- | --- | --- | --- | --- |
|  | **Platform** | **Inclusion criteria** | | | **SNPs met**  **QC criteria** | **Software** | **Inclusion criteria** | | **SNPs in**  **meta-analysis** | **λ_GC_** | **Software** | **covariates** |
|  |  | **MAF** | **Call rate** | **P HWE** |  |  | **MAF** | **Imputation quality** |  |  |  |  |
| NHS BrC | Illumina 550k | ≥1% | ≥90% | - | 528,173 | MACH | >2% | Rsq ≥0.5 | 2244803 | 1.00 | ProbABEL | age, BrC case-control status, top 4 eigenvectors |
| NHS CHD | Affymetrix 6.0 | ≥2% | >98% | ≥10^-4^ | 721,316 | MACH | >2% | Rsq ≥0.5 | 2196994 | 1.01 | ProbABEL | age, CHD case-control status, top 3 eigenvectors |
| NHS T2D | Affymetrix 6.0 | ≥2% | >98% | ≥10^-4^ | 704,409 | MACH | >2% | Rsq ≥0.5 | 2186671 | 1.01 | ProbABEL | age, T2D case-control status, top 3 eigenvectors |
| NHS KS | Illumina 610Q | ≥1% | ≥95% | ≥10^-5^ | 546,344 | MACH | >2% | Rsq ≥0.5 | 2244671 | 1.00 | ProbABEL | age, KS case-control status, top 4 eigenvectors |

**Supplementary Table S3. Sample quality control**

| **Study** | **Sample Quality Control** | | **Sample size included** |
| --- | --- | --- | --- |
|  | **Call rate** | **Other exclusion criteria** |  |
| NHS BrC | >90% | -duplicates and first/second degree relatives  -ancestry outliers  -missing phenotype information | 2,280 |
| NHS CHD | >98% | -sex discrepancy with genetic data from X-linked markers  -duplicates and first/second degree relatives  -ancestry outliers  -heterozygosity  -missing phenotype information | 1,135 |
| NHS T2D | >98% | -sex discrepancy with genetic data from X-linked markers  -duplicates and first/second degree relatives  -ancestry outliers  -heterozygosity  -autosomal chromosome abberations  -missing phenotype information | 3,084 |
| NHS KS | ≥95% | -duplicates and first/second degree relatives  -ancestry outliers  -missing phenotype information | 490 |

**Supplementary Table S4. Percent of variance* in depression phenotypes explained by variables associated with depression in the NHS full cohort, restricting to women with non-missing 2004 CESD-10 and 14-year long-term average depression scores (N=73,897)**

| **Variables** | **CESD-10 score** | **14-year average depressive measure** | **Relative improvement**  **in % of variance explained** |
| --- | --- | --- | --- |
| Cigarette smoking | 0.49 | 0.68 | 39% |
| Physical activity | 2.24 | 4.49 | 100% |
| Husband highest education | 0.43 | 0.48 | 12% |
| Father's occupation when participant was 16 years old | 0.13 | 0.16 | 23% |
| Phobic anxiety** | 7.68 | 8.73 | 14% |

* The model included each variable associated with depression adjusting for age. Percent of variance explained by the variable is the r^2^ from the linear regression of depression score on each variable plus age minus the one with age alone, times 100. The relative improvement in percent of variance explained is the difference of r^2^ between using 14-year long-term average depressive measure and using the CESD-10 in 2004 divided by the r^2^ using CESD-10 in 2004.

** The average of phobic anxiety scale of the Crown Crisp Experimental Index (CCI) between 1988 and 2004

**Supplementary Table S5. Meta-analysis of percentage of variance explained in depression phenotype in NHS by the candidate gene polygenic scores in the leave-one-substudy-out analysis (N=6,989)**

| Cumulative p-value thresholds for selecting SNPs | | | Non-overlapping P-value thresholds for selecting SNPs | | |
| --- | --- | --- | --- | --- | --- |
| P_training_^*^ threshold | Percentage of variance explained | *p*-value | P_training_^*^ threshold | Percentage of variance explained | *p-*value |
| p < 0.00001 | – | – | 0-0.00001 | – | – |
| p < 0.0001 | – | – | 0.00001-0.0001 | – | – |
| p < 0.001 | – | – | 0.0001-0.001 | – | – |
| p < 0.01 | 0.1 ^†^ | 0.647 | 0.001-0.01 | 0.1 ^†^ | 0.647 |
| p < 0.1 | 0 | 0.172 | 0.01-0.1 | 0 | 0.207 |
| p < 0.2 | 0.1 | 0.002 | 0.1-0.2 | 0.1 | 0.004 |
| p < 0.3 | 0.2 | 0.001 | 0.2-0.3 | 0.1 | 0.076 |
| p < 0.4 | 0.1 | 0.003 | 0.3-0.4 | 0 | 0.760 |
| p< 0.5 | 0.1 | 0.001 | 0.4-0.5 | 0.1 | 0.101 |

^*^ Training set: remaining 3 NHS substudies except the target sample

^†^ N=1,625 (NHS T2D and BrCa substudies were not included because no SNP was in p<0.01 threshold range)

**Supplementary Figure S1. Quantile plot of polygenic scores (PS) on 14-year long-term average composite depression phenotype**
